# Investigating the rate of different ovarian response in in vitro fertilization cycles based on estrogen receptor beta +1730 polymorphism: A cross-sectional study

**DOI:** 10.18502/ijrm.v13i7.7368

**Published:** 2020-07-22

**Authors:** Elham Parsa, Seyed Mehdi Hoseini, Seyedeh Mahdieh Namayandeh, Zhima Akhavansales, Mohammad Hasan Sheikhha

**Affiliations:** ^1^Medical Biotechnology Research Center, Ashkezar Branch, Islamic Azad University, Ashkezar, Yazd, Iran.; ^2^Biotechnology Research Center, International Campus, Shahid Sadoughi University of Medical Sciences, Yazd, Iran.; ^3^Afshar Research and Development Center, Shahid Sadoughi University of Medical Sciences, Yazd, Iran.; ^4^Research and Clinical Center for Infertility, Shahid Sadoughi University of Medical Sciences, Yazd, Iran.

**Keywords:** Gene, Polymorphism, Estrogen receptor gene, Infertility.

## Abstract

**Background:**

The response to ovarian stimulation is different among women referring for assisted reproductive techniques. This difference could be due to different genotypes in genes related to reproduction such as estrogen receptor beta (*ERβ or ESR2*) gene.

**Objective:**

In the present study, we explored the rate of *ESR2* gene polymorphism in infertile women undergoing IVF treatment with different ovarian response to ovulation induction.

**Materials and Methods:**

A cross-sectional study was performed among 91 infertile women. The relationship between genotype distribution of the +1730 G/A polymorphism in the *ESR2 *gene (rs4986938) and the mean number of follicles and oocytes, their mean ratio, mean number of embryos, mean size of the follicles and pregnancy rates were measured. The *ESR2 *gene +1730 G/A polymorphism were identified by the amplification-refractory mutation system-polymerase chain reaction.

**Results:**

Genotypes GG, GA, and AA of the *ESR2 *gene presented frequencies of 27.5%, 67%, and 5.5%, respectively, in the infertile women. The results of the study showed that the mean number of follicles and oocytes, their mean ratio, mean number of embryos, mean size of the follicles, and pregnancy rates are not related to different genotypes.

**Conclusion:**

According to the endocrine and paracrine factors which are involved in the ovulation induction and maturation of oocytes, more studies with higher number of participants are required to confirm the results of the present study; in addition, further studies are required to find out other gene polymorphisms affecting estrogen receptor efficacy in the infertile women.

## 1. Introduction

Based on academic definition, infertility is a failure to achieve clinical pregnancy after 12 months of regular unprotected sexual intercourse (1). It is estimated that infertility affected more than 70 million couples worldwide especially in developing countries (2, 3). However, it seems that with advanced technology to help infertile couples, the incidence of infertility is decreasing, for example it was shown that in the United States of America this incidence declined from 11.2% in 1965 to 9% in 2007. Besides, it was as high as 30% in Sub-Saharan Africa, about 12% in some European communities, and nearly 8.5% in Canada (4-6). The prevalence of infertility was highest in central/eastern Europe, north Africa/Middle East, sub-Saharan Africa and south/central Asia (7). There are some data about the infertility rate in different regions of Iran like Shiraz with infertility rate of 11.1%, west of Tehran with 12%, Yazd with 5.52% and even in one study, a history of lifetime primary infertility was present in 24.9% of the Iranian women (5, 8). Parsanezhad and colleagues showed that “the average prevalence rate of life time infertility is 10.9% in Iran and 3.3% of the population has current infertility. The reported causes of infertility in the couples include tubal and pelvic pathology (35%), ovulatory dysfunctions (15%), male problems (35%), unexplained infertility (10%), and unusual problems (5%)” (2).

Female factors infertility is due to several factors, including abnormal hormones and its effect on the performance of female genital tract (9). Estrogen plays an important role in fetal development, the development of secondary sexual characteristics, fertility cycle, and maintaining pregnancy (10). In addition, estrogen regulates the growth and differentiation of endometrial cells (11). Estrogens are steroid hormones that play a vital role on both the female and male reproductive systems (12). In addition, they are important in the cardiovascular system and in the maintenance of bone tissue (12, 13). Estrogens imply their actions through a ligand-activated transcription factor, the estrogen receptor (ER) (14), which has two isoforms; ERα and ERβ, encoded by different genes (15). *ERβ* has been expressed primarily in the ovary, colon, fallopian tube, lung, adipose, kidney, heart, bladder, adrenal, testis, prostate, brain and bone, whereas it has been seen that *ERα* are predominantly detected in testis, liver, ovary, uterus, mammary gland, adipose, bone, heart, and brain (pituitary gland) (13, 16-18).

The *ERβ* gene also named as *ESR2* gene is located on chromosome 14q22-24 (19). Two common single nucleotide polymorphisms was displayed by screening of the coding region and part of the 5' and 3' regions of the *ESR2* gene: G/A exchange at nucleotide 1730 in the 3' untranslated region in exon 8 (rs4986938) and a silent 1082 G/A transition in exon 5 (14, 19, 20). Both polymorphisms have been overrepresented in ovulatory dysfunctions (19).

Bianco and colleagues found that the +1730 G/A polymorphism in the *ESR2* gene was associated with an increased risk of developing endometriosis in infertile women (21).

However, studies on genetic variants of *ESR2* with respect to female infertility are still controversial. Accordingly, our aim was to investigate the impact of the *ESR2* gene +1730 G/A polymorphism on ovarian response in IVF cycle.

## 2. Materials and Methods

This was an analytical research done with cross-sectional method. In total, 91 eligible samples referred to the Research and Clinical Center for Infertility, Yazd, Iran, from February 2014 to January 2015, were selected consecutively. The couples with male infertility and endometriosis were excluded from the study. The age range of the studied samples was 21-43 yr with the mean age of 32.4 ± 4.5 yr. The duration of infertility was from 1.5 to 21 year with an average of 6.4 ± 2.7 year. The cause of infertility in the selected population was ovarian disorders with 33.3%, disorders of the fallopian tube 14%, polycystic ovary syndrome 26.9%, unknown disorders 9.12%, and abnormalities in uterine 2.2%, whereas 10.8% were related to two-factors of infertility.

For ovulation induction, all participants were treated with antagonist protocol (with prescribed Gonal, Cetrotide, HMG, and hCG) and other information such as the number of follicles, ovum, and embryos as well as chemical pregnancy outcome after registration by the relevant specialist was recorded from the patient.

In this study, amplification refractory mutation system-polymerase chain reaction method was used to determine the *ESR2* gene +1730 G/A polymorphism. In order to design the required primers, the sequence surrounding the *ESR2* gene was first taken referring to gene information bank located in the National Center for Biotechnology Information online databank (http://www.ncbi.nlm.nih.gov). Subsequently, suitable primers were designed by using the Primer3 tool (http://frodo.wi.mit.edu/cgi bin/primer3) and the final analysis was performed using the Oligo 7 software package (Table I). Based on the PCR results, three different alleles AA, GG, and AG were detected.

**Figure 1:**
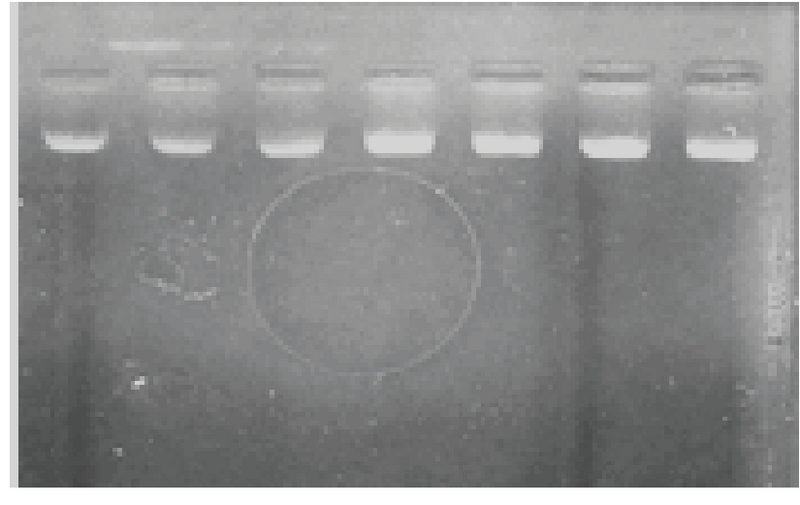
The products of DNA extraction were run on 1% agarose gel. DNA samples were not broken as was visualized under UV light.

DNA extraction was performed according to the Gene All Kit, Exgene Blood SV mini, 250p, South Korea. After extraction, the products were run on 1% agarose gel to verify the results and the quality of the genomic DNA (Figure 1).

**Figure 2:**
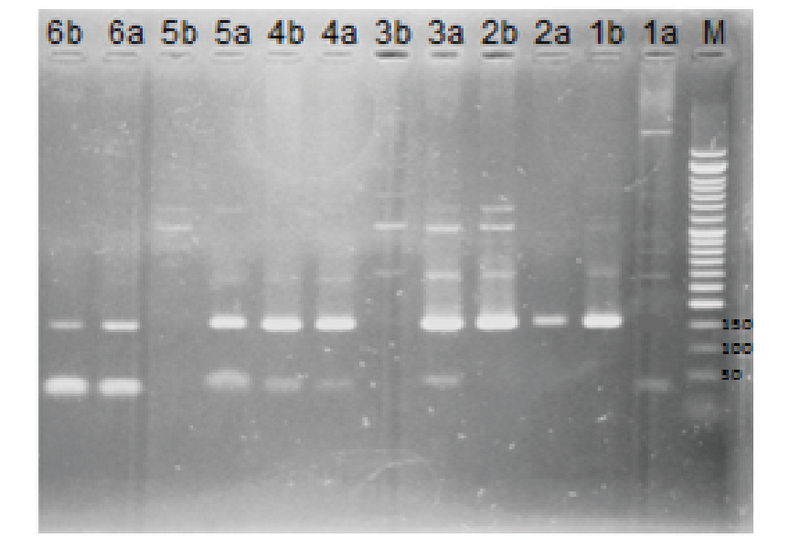
Agarose gel electrophoretic analysis of the *ESR2* gene +1730 G/A polymorphism: Lane (M) represents the molecular marker (DNA molecular weight marker). Lanes (1a and1b) represent the AA genotype showing one band at 157 bp. Lanes (2a and 2b, 4a and 4b, 6a and 6b) represent GA genotype that is presented by two bands at 157 bp. Lanes (3a and 3b, 5a and 5b) represent the GG genotype showing one band at 157 bp.

Then, the genomic DNA was analyzed for intended polymorphism using amplification refractory mutation system-polymerase chain reaction method. The overall mixed solution of the used reaction was Taq DNA Pol 2X Master Mix Red from Ampliqon Company. The solution containing compounds required for PCR include a variety of dNTP (0.4 mM), the enzyme Taq polymerase (0.2 units/μl master mix), and MgCl${}_{2}$ (3 mM); 1X solution of this reaction mixture was used for a PCR reaction. The total volume of each tube was 20 μl which contained 10 μl Master mix, 2 μl Primer, 4 μl genomic DNA, and 4 μl of double distilled water. Amplification was set by PCR during 35 consecutive cycle and binding temperature of primers to 65°C for 1 min. Denaturing and elongation processes in all cycles were done at 94°C and 72°C for 30 sec and 1 min, respectively. Before the beginning of the cycle, denaturing of total genomic DNA was performed for 10 min at 94°C. Moreover, after the completion of the all cycles, elongation period for 10 min at a temperature of 72°C was considered for completing the unfinished chains. After the completion of PCR reactions, the products were run on 2% agarose gels alongside a 50 bp marker. After electrophoresis and a dying step with ethidium bromide, bands were observed using a UV Gel Doc apparatus (Figure 2).

**Table 1:** The sequence of the primers used in the study


**Primers**	**Sequence 5'-3'**
**F1**	5'- GGCCCACAGAGGTCACAG-3'
**F2**	5'- GGCCCACAGAGGTCACAA-3'
**R**	5'-CTTCCTCACACCGACTCCTG-3'

### Ethical consideration

The study was approved by the ethical committee of Yazd Reproductive Sciences Institute. Written consents were taken from all participants after informing them about the aims and method of the study.

### Statistical analysis

Data was analyzed using SPSS software (Statistical Package for the Social Sciences, version 16.0, SPSS Inc, Chicago, Illinois, USA). Chi- square test and *t* test were used for evaluating the differences between groups. Logistic regression method was used for confounder adjournment.

## 3. Results

Our results indicated that most frequent polymorphism was AG with 67%, while the GG polymorphism was 27.5% and AA polymorphism was 5.5%. According to the X2 test and p = 0.96, there was no statistically significant relationship between the *ESR2* gene +1730 G/A polymorphism and the result of pregnancy (Table II).

In each of the ART cycles, the number of ovum, embryos, and the ratio of ovum to follicle were calculated, and there was no statistically significant relationship between these variables and the *ERβ* gene +1730 G/A polymorphism status (Table III).

The association of polymorphisms with the number of follicles was examined. For this purpose, the number of follicles was classified in three groups: < 5, 5-15, and > 15. Considering that the majority of the follicle was with GA polymorphism 60.5% and p = 0.55, there was no statistically significant relationship between the number of follicles and the *ESR2* gene +1730 G/A polymorphism (Table IV).

Based on the linear regression analysis, age and serum estrogen levels were important independent factors for follicle number in stimulation of ovulation (Table V).

Based on the logistic regression analysis of follicle numbers and other factors such as age, duration of infertility, serum estrogen density, genotypes, and important infertility factors, the result of pregnancy is identified (Table VI).

**Table 2:** The result of pregnancy based on the *ESR2* gene +1730 G/A polymorphism


**Result of pregnancy** **Polymorphisms**	**GG**	**GA**	**AA**
**Positive **	6 (26.1)	15 (25.4)	1 (20)
**Negative**	17 (73.9)	44 (74.6)	4 (80)
**Total**	23	59	5
Data presented as n (%); p-value = 0.96; Chi-square test was used			

**Table 3:** The number of ovum, embryos, and the ratio of ovum to follicle based on the *ESR2* gene +1730 G/A polymorphism


**Variable** **Polymorphisms**	**GG**	**GA**	**AA**	**P-value**
**Number of embryos**	6.2 ± 5.3	7.6 ± 6.3	6 ± 4.9	0.5
**Number of ovum**	16.6 ± 8.3	16.3 ± 9.4	19 ± 8.1	0.8
**Ovum to follicle ratio**	0.75 ± 0.24	0.73 ± 0.26	0.78 ± 0.25	0.9
Data presented as Mean ± SD; *t* test was used				

**Table 4:** Relationship between the number of follicles and polymorphism


**Number of follicles** **Polymorphisms**	**GG**	**GA**	**AA**	**P-value**
**< 5**	2 (50)	2 (50)	0 (0)	
**5-15**	9 (20.9)	32 (74.4)	2 (4.7)	
**> 15**	14 (32.6)	26 (60.5)	3 (7)	0.55
Data presented as n (%), Chi-square test was used				

**Table 5:** Follicle number determinants based on linear regression analysis using enter model


**Effective factors**	**Linear regression**	**Standard deviation**	**T**	**P-value**
**Age**	-0.45	0.16	-2.7	0.0001
**Duration of infertility**	0.055	0.15	0.34	0.73
**Serum estrogen density**	0.002	0.000	7.8	0.0001
**Genotypes**	-0.5	1.3	3.37	0.7
According to linear regression analysis, the age and serum estrogen density determines the follicle number as an independent factor				

**Table 6:** Pregnancy determinants based on logistic regression analysis using enter model


**Effective factors**	**Odds ratio**	**Standard deviation**	**P-value**
**Age**	0.96	0.065	0.54
**Duration of infertility**	1.02	0.06	0.74
**Serum estrogen density**	1	0.000	0.11
**Genotypes**	1.3	1.2	0.8
**Infertility cause**	0.97	0.21	0.89
**Follicle number**	1.08	0.43	0.04
According to logistic regression analysis, the number of follicles determines the outcome of pregnancy as an independent factor			

## 4. Discussion 

Infertility is caused due to several factors, including hormones and their effects on the performance of female genital tract (9). One of the two women's sex steroid hormones is estrogen which is released by the ovaries (12). Estrogen plays an important role in fetal development, the development of secondary sexual characteristics, fertility cycle, and maintaining pregnancy (10). In addition, estrogen regulates the growth and differentiation of endometrial cells (11). ER is divided into alpha (ERα) and beta (ERβ) types (15). In a research, removing beta receptors in mice leads to low ovum production and eventually infertility, which is due to the defects in ovarian tissue (22). In another research on *ERβ* gene knockout mice, it was confirmed that ERβ is essential for normal ovulation but not for sexual differentiation, reproduction, and lactation (23). Practical importance of the *ESR2* gene +1730 G/A polymorphism is not clear as it does not lead to amino acid changes in the ERβ protein. However, it is possible that the polymorphism is associated with other regulatory sequence changes that may affect gene expression and its function (11).

Recently, much attention has been given to personalized medicine. The patient's pharmacogenetics is important in the treatment of diseases, therefore depending on the patient's drug metabolism in the body, medication and dosage is determined. So, in infertility treatment, the patient's genetics effects in response to treatment should be considered. According to the mentioned studies, it has been shown that the *ESR2* gene +1730 G/A polymorphism plays a role in ovulation and therefore we selected the polymorphism to review its effects on the outcomes of IVF. To study the role of genetics response to treatment in infertile patients in this study, the *ESR2* gene +1730 G/A polymorphism role in the response to treatment of 91 infertile women were analyzed. Considering the need to remove the confounding factors in these patients for studying genetic effects on the response to treatment, the patients whose infertility were related to male factors and endometriosis were excluded. Also, because the purpose of this study was to evaluate the response of different polymorphism to the treatment in infertile patients, the normal cases were not considered.

In Seleem and co-workers' study, GG genotype was more frequent than the other genotypes, while in the current study, the frequency of GA genotype was more than other genotypes. This difference could be due to racial differences in the two groups or the number of the sample (15). To evaluate the response of patients to treatment in this study, the number of follicles and number of ovum after stimulation of ovulation as well as the ratio of ovum to follicles were considered. This ratio is important in the sense that some follicles may be empty without any ovum. The results of the ratio of ovum to follicles in all groups was equal, so, there was no significant difference; however, in the current study, the number of follicles in the GA group was more, but this difference was not statistically significant.

Overall, in the current study, GA group achieved the highest percentage of pregnancies (25.4%) while AA group's was the lowest (20%). Although there was no similar study in this region to our knowledge, but we can refer to Sheikhha and *colleagues'* study about ER alpha gene that showed that 40% of heterozygote Pp group achieved pregnancy which was the highest success rate while the lowest rate of pregnancy was in pp group with 20% (24). So, the highest responses to treatment in both studies were in heterozygous genotype group. It could be due to the factor that for the success of IVF, the estrogen levels should be at certain level, while the smaller or greater amounts of it will have negative consequences in response to the treatment.

In general, the observed differences in the number of ova, follicles, and the ratio of embryos between this study and others' might be due to racial and genetic differences between the groups. In addition, other factors such as treatment protocol, the profile of patients, their age, and so on, can be effective. Because the statistical analysis of our results was not significant, it should be noted that based on linear regression analysis, age and serum levels of estrogen are the independent factors for number of follicle in stimulating ovulation. Moreover, according to logistic regression analysis, the number of follicles determines the outcome of pregnancy, as an independent factor, regardless of other factors such as age, duration of infertility, serum density of estrogen, genotype, and infertility factor.

## 5. Conclusion

According to the relatively low percentage of treatment responses to IVF cycles, more attention is needed to focus on pharmacogenetics and patient's individual response. This study investigated the importance of a gene involved in human reproduction. Based on statistical analysis, the number of follicles is related to the outcome of pregnancy while age and estrogen are the effective factors on follicle numbers. For further study the role of the *ESR2* gene +1730 G/A polymorphism in infertility, the prevalence of the polymorphism is necessary to be evaluated in normal populations.

If various differences in infertile women are identified by further investigations and determination of polymorphisms in different genes, proper planning can be done to choose effective agents in the treatment of each individual according to the genetic background. This will eventually lead to an increase in pregnancy rates in infertile patients, preventing excessive use of drugs and avoiding to give false hope to infertile couples.

##  Conflict of Interest

There was no conflict of interest in this study.
